# Studying lack of awareness of cognitive decline in neurodegenerative diseases requires measures of both anosognosia and denial

**DOI:** 10.3389/fnagi.2023.1325231

**Published:** 2024-01-08

**Authors:** George P. Prigatano, Sydney Russell, Tiffany M. Meites

**Affiliations:** Department of Clinical Neuropsychology, Barrow Neurological Institute, St. Joseph’s Hospital and Medical Center, Phoenix, AZ, United States

**Keywords:** mild cognitive impairment, early Alzheimer’s dementia, anosognosia, denial, case examples

## Abstract

The cause(s) of lack of awareness of cognitive decline in neurodegenerative diseases can be multifactorial. Yet neurologically oriented research on anosognosia of cognitive decline almost exclusively assumes that the underlying disturbance of neuro-networks that support various cognitive functions accounts for the reduced self-awareness. Cultural and psychosocial factors, including the person’s emotional state, however, can contribute to the underreporting or avoidance of admitting to cognitive impairments in neurodegenerative diseases. Research on the causes of lack of awareness of cognitive decline in neurodegenerative disorders needs to include these variables. We briefly present two case examples of underreporting or “unawareness” of memory difficulties in persons with mild cognitive impairment (MCI) (or minor neurocognitive disorder). One presented with classic anosognosia for memory impairment, while the other initially reported no memory impairment but later admitted to “denying” her memory difficulties secondary to anxiety. Based on these patients’ clinical presentations and available research, we suggest three potential screening items that may help identify probable denial of memory impairments when studying anosognosia in MCI.

## Introduction

Clinicians have long recognized that persons who demonstrate cognitive decline in conjunction with various neurodegenerative diseases frequently underreport their symptoms (Rowe and Katzman, 1992). The factors underlying this underreporting are not always obvious. Ample evidence associates underlying brain pathology/dysfunction with the clinical phenomenon of anosognosia in mild cognitive impairment (MCI) and Alzheimer’s disease (AD) (Mondragón et al., 2019). Depending on the neuroimaging metrics used and methods for assessing the lack of awareness, numerous and diverse findings have been reported. Multiple brain region disruptions appear to contribute to anosognosia in MCI of the amnestic type () and AD, including midline frontal regions, especially on the right, and complex disruptions of the Default Mode Network (DMN) (Mondragón et al., 2019). More recent research has reported different patterns of neuroconnectivity based on the domains of anosognosia assessed in early AD, but “total anosognosia scores” have been particularly linked with disruption of frontal–parietal networks (Valera-Bermejo et al., 2022, pg. 1). Gagliardi and Vannini (2022) point out “…that amyloid and tau accumulation patterns in the brain overlapped with the DMN” (pg. 3); they additionally indicated that amyloid levels have been positively correlated with some measures of impaired awareness of memory in persons with MCI and early dementia. It also has been reported that anosognosia in MCI and AD is common. One report suggests that “severe anosognosia” was present in 55.6% of persons with AD and 9.5% with MCI (Mak et al., 2015). Cognitive dysfunction and apathy are often associated with the presence of anosognosia within this patient population (Mak et al., 2015).

While many statistically significant findings have been observed by numerous investigators between biological markers of AD (Gagliardi and Vannini, 2022) and impaired awareness and neuropsychological markers in MCI and AD (Orfei et al., 2010), the correlations are typically mild to moderate in size. This suggests at least two possibilities. First, the most sensitive biological and neuropsychological correlates of impaired self-awareness (ISA) remain unidentified. Second, some patients who have ISA of their memory impairments may actually be denying those impairments as a psychological method of coping rather than demonstrating true “anosognosia” (Prigatano and Sherer, 2020). The second possibility has not been adequately considered when exploring the relationship between various neuroimaging findings and measures of anosognosia.

A repeated clinical and research observation has been that non-neurological factors may contribute to failures of underreporting memory impairments. Weinstein and Kahn (1955) were perhaps the first contemporary investigators to provide convincing clinical evidence for this point of view. Gainotti (1975), following up on Weinstein and Kahn’s (1955) observations, assessed how the person’s attitude toward their health, work and illness might influence the reporting of memory impairments during the early stages of dementia. He also attempted to determine how cultural factors might influence the patient’s subjective reports regarding their memory. He noted that “denial” or “lack of awareness” was not strongly correlated with the degree of memory degradation. Rather he found that the person’s pre-existing attitudes toward illness contributed to the presence of “denial.” If the person had a tendency to regard “illness as imperfection,” they were likely to deny their decline in memory (Gainotti, 1975, pg. 103). Quite interestingly, he noted that “denial among the patients of the highest classes” (pg. 103) was also more frequent. In the first cross-cultural study of this phenomenon, Gainotti (1975) found that Swiss patients had a higher frequency of denial than Italian patients did.

In a large cross-cultural study, Mograbi et al. (2012) reported cultural differences in the prevalence of unawareness of memory impairment in persons with dementia. The highest frequency of unawareness was found in India (81.2%) in comparison to Latin America (72.0%) and China (63.5%)” (pg. 935). Compatible with Gainotti’s (1975) earlier observations, Mograbi and his colleagues propose that “unawareness was significantly associated with educational level—with an increase in the frequency of unawareness in the highest educational group” (pg. 935). These investigators also noted that the emotional state of the person is at times related to the presence of unawareness. For example, unawareness was significantly associated with depression, but not in India. The authors concluded “…unawareness should be seen not only as a common neurobiological feature of dementia, increasing with severity of dementia, but also as a phenomenon influenced by social and cultural factors” (pg. 931).

The failure to assess the person’s emotional state when obtaining subjective reports regarding their clinical condition, including their awareness of cognitive decline, can potentially obscure important research findings on anosognosia. In this regard, it important to distinguish individuals who appear truly unaware of their cognitive impairments, as in the case of anosognosia, from those who are aware but deny their impairments to avoid anxiety or perhaps further depression/anger over their clinical condition (Prigatano, 2014). Patients with anosognosia of cognitive impairments often appear apathetic or indifferent (Prigatano et al., 2011, 2014; Mak et al., 2015; Bivona et al., 2019) rather than anxious or depressed when talking about their cognitive impairments. In contrast, persons who demonstrate denial often appear ill at ease when speaking about their cognitive limitations, even becoming anxious and/or angry when discussing them with family members or a clinician (Prigatano et al., 2020; Prigatano and Sherer, 2020).

In light of these considerations, the aim of this paper is to explore, via case examples, how a person with MCI with anosognosia may differ from a person with MCI who denies their memory impairments. We briefly describe two persons with MCI who jointly reported experiencing no memory impairments beyond the normal aging process. One person (Patient #2) later admitted being aware of her memory difficulties but stated that she did not want to admit them to herself or others out of anxiety – a case of denial. The second person (Patient #1) persisted with his statements of not experiencing any memory impairments and showed no signs of anxiety – a case of anosognosia. We briefly compare their clinical presentations, neuropsychological test findings, and significant others’ reports of their behaviors to explore possible emotional/behavioral markers that might help screen persons with probable denial when studying anosognosia for memory impairments in MCI and AD.

## Case reports: anosognosia versus denial of memory impairments in MCI

Patient #1 was a 72-year-old, right-handed, Caucasian male, with 12 years of education. He stated that he did not experience any unusual difficulty with memory but recognized that his wife had a different opinion. He was willing to answer all questions during the interview but appeared perplexed as to why he needed to undergo a neuropsychological examination. He appeared to present as a clear case of anosognosia for his memory impairment.

Patient #2 was a 69-year-old, right-handed, Caucasian female, with 12 years of education. This patient did not report any memory difficulties, although family members reported an insidious development of memory difficulties over the preceding 2 years. She fully understood the reason for the evaluation. After the examination was complete, she admitted to denying her memory difficulties secondary to anxiety. She presented as a case of (mild) denial of memory impairment.

### Patients’ qualitative and quantitative neuropsychological test performance

While many researchers rely on brief screens to assess cognitive functioning, detailed neuropsychological evaluation was completed in order to comprehensively compare the individuals in this paper. On the Wechsler Adult Intelligence Scale, Fourth Edition (WAIS-IV; Wechsler, 2008), levels of performance were very similar on three of the four composite scores for both patients (Table 1). Patient #1 performed in the impaired range on the Processing Speed Index (PSI) Score, while Patient #2 performed in the average range. On the Wechsler Memory Scale, Fourth Edition (WMS-IV; Wechsler, 2009), both patients performed in the impaired range with quite similar levels of performance (Table 1). The same was true when comparing their performances on the Rey Auditory Verbal Learning Test (RAVLT; Lezak et al., 2004) and the Brief Visual Memory Test, Revised Form (BVMT-R; Benedict et al., 1996; Table 1).

**Table 1:** Neuropsychological test performance and reports on the Patient Competency Rating Scale in an MCI patient with denial (Patient #2) versus with anosognosia (Patient #1).

Name of test		Patient 1	Patient 2
WAIS-IV (composite indices_1_)	Verbal Comprehension Index (VCI)	95	93
	Perceptual Reasoning Index (PRI)	90	100
	Working Memory Index (WMI)	92	95
	Processing Speed Index (PSI) Full	76	108
	Scale Intelligence Quotient (FSIQ)	84	98
WAIS-IV (Subtests_2_)	Similarities	8	10
	Vocabulary	9	7
	Information	10	9
	Block Design	7	9
	Matrix Reasoning	11	10
	Visual Puzzles	7	11
	Digit Span	9	9
	Arithmetic	8	9
	Symbol Search	4	11
	Coding	7	12
RAVLT	Total 1–5	23/75_3_, T = 38_4_	27/75_3_, T = 20_4_
	Long Delay	1/15_3_, T = 32_4_	2/15_3_, T = 17_4_
	Recognition	N/A_5_	11/15_3_, T = 49_4_
BVMT-R	Total 1–3	9/36_3_, T = 29_4_	7/36_3_, T = 25_4_
	Delayed Recall	0/12_3_, T = <20_4_	1/12_3_, T = 20_4_
	Recognition	2/6_3_, 2f + _6_	5/6_3_, 2f + _6_
WMS-IV LM	Immediate Recall	24/53_3_, ss = 7_2_	21/53_3_, ss = 6_2_
	Delayed Recall	0/39_3_, ss = 1_2_	2/39_3_, ss = 2_2_
	Recognition	15/23_3_, 10–16%ile_7_	14/23_3_, ≤2%ile_7_
BNT		47/60_3_, T = 44_4_	53/60_3_, T = 46_4_
Verbal fluencies	COWAT (Phonemic fluency)	24 words, T = 38_4_	44 words, T = 53_4_
	Semantic fluency (category [animals])	5 words, T = 17_4_	17 words, T = 48_4_
TMT	Part A	T = 37_4_, 60″ _8_, 0e_9_	T = 66_4_, 20″_8_, 0e_9_
	Part B	T = 33_4_, 182″_8_, 0e_9_	T = 65_4_, 54″_8_, 0e_9_
WCST-64	Total errors	45, T = 29_4_	25, T = 43_4_
	Perseverative errors	35, T = 29_4_	11, T = 49_4_
	Categories completed	0, 2-5%ile_7_	2, 11-16%ile_7_
HFTT (mean speed)	Dominant right hand	46.8, T = 46_4_	44.1, T = 51_4_
	Non-dominant left hand	40.1, T = 45_4_	37.6, T = 50_4_
PCRS	Patient’s form	128/150_10_	131/150_10_
	Relative’s form	98/150_10_	119/150_10_

WAIS-IV, The Wechsler Adult Intelligence Scale, Fourth Edition; RAVLT, Rey Auditory Verbal Learning Test; BVMT-R, Brief Visuospatial Memory Test, Revised Version; WMS-IV, Wechsler Memory Scale, Fourth Edition; BNT, Boston Naming Test; Verbal fluencies, (COWAT, Controlled Oral Word Association Test; Category fluency [Animal Naming]); Trail Making Test, Parts A and B; WCST, Wisconsin Card Sorting Test, 64-Card Version; HFFT, Halstead Finger Tapping Test; PCRS, Patient Competency Rating Scale, PCRS-R, PCRS Patient and Relative’s Forms. ^1^Age-adjusted standard scores. ^2^Age-adjusted scaled scores. ^3^Number of words recalled of the total possible amount. ^4^T = (T-score): Standardized score in which 50 represents the mean level of performance with a standard deviation of 10 points. ^5^N/A = Test not administered due to the patient’s clinical condition. ^6^f + =Number of false positives made. ^7^%ile = Percentile ranking. ^8^X” = Seconds to complete the task. ^9^e = Number of errors made. ^10^Total raw score.

Patient #1 had significant difficulty with a category word fluency task (Lezak et al., 2004), but Patient #2 did not (Table 1). Both patients had a comparable confrontational naming performance on the Boston Naming Test (BNT; Kaplan et al., 1983; Table 1). The Trail Making Test, Part B (TMT B; Lezak et al., 2004) was significantly more challenging for Patient #1 than for Patient #2 (Table 1). Patient #1’s number of perseverative errors on the Wisconsin Card Sorting Test, 64 Card Version (WCST-64; Heaton et al., 1993) was in the impaired range, but this was not true for Patient #2 (Table 1). There was no difference in the mean speed of finger tapping (Halstead, 1947) between the two patients (Table 1). Collectively, these psychometric findings suggest both patients had mild cognitive impairments in multiple domains.

### Patients’ emotional reactions when performing neuropsychological tests

Both patients were cooperative during their three-to-four-hour neuropsychological consultation/examination. While each commented that they had difficulty performing some of the memory tasks, neither appeared concerned about their performance. They stated that, given their age, they would expect to have difficulty completing some of the memory tasks. Neither one appeared defensive. Instead, both appeared dismissive of their performance failures on memory tests.

### Patients’ ratings of their functional abilities compared to a reliable relative’s ratings

The patients’ self-reported levels of competency did not differ and fell within the normal range for their age on the Patient Competency Rating Scale (PCRS; Prigatano et al., 1986; Table 1). Both reported no difficulties in daily functioning, including their memory functions. However, the spouse of Patient #1 described him as less functional in everyday life than what the patient self-reported. The spouse of Patient #2 also reported less functional capacity than what the patient self-reported, but the patient’s level of daily functioning was much higher than for Patient #1 (i.e., 119/150 versus 98/150). Specifically, the spouse of Patient #1 noted he could not handle finances or recognize when something he said or did upset someone else, while the spouse of Patient 2 stated no difficulties in either area.

### Patients’ level of concern over their impairments and their emotional reactions when talking about their difficulties

When asked to rate the patient’s level of concern regarding difficulties in everyday life on a scale from zero (no concern) to 10 (extreme concern), Patient #1’s spouse rated him as a one. Patient #2’s spouse rated her as a four. When asked about the level of anxiety or anger/agitation the patient typically shows when talking about their difficulties, Patient #1’s spouse rated him as a two; Patient #2’s spouse rated her as an eight.

## Implications for screening for denial when studying anosognosia in MCI

**Figure 1:**
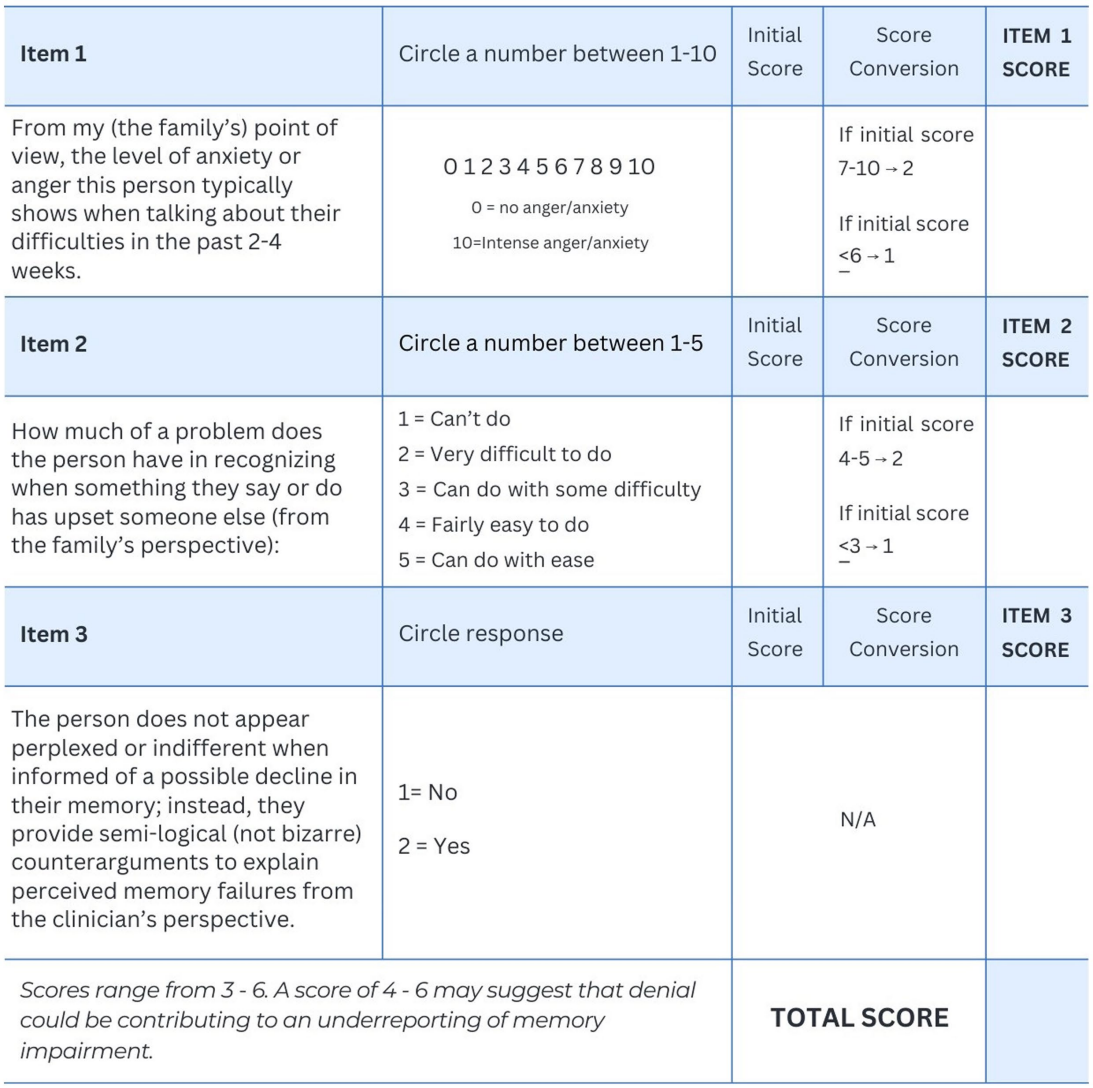
Screening measure for denial in persons with suspected memory impairments.

Since denial is a hypothetical construct that attempts to explain an apparent emotional reaction to impairments/disabilities (Lazarus, 1983), we suggest three emotional/behavioral markers for screening purposes in light of differences observed in these two patients. The first proposed marker is elevated anxiety or anger displayed by the patient when discussing their limitations with a significant other or a clinician. Asking family members to rate this emotional feature of the person’s behavior may be a potentially useful screening item (see Figure 1, Item #1).

The second proposed marker is the capacity of the person to perceive when they have said or done something that upsets another person. This was an item on the PCRS-R that clearly differentiated the two patients. Individuals with “true” anosognosia frequently have difficulty performing this task in daily life (Prigatano, 2014). The second screening item attempts to asses this ability via relatives’ reports. Item #2 is adapted from the PCRS (Prigatano et al., 1986) and is rated on a five-point scale ranging from “cannot do” to “can do with ease.” (see Figure 1).

The third proposed emotional/behavioral marker of severe denial is active resistance to “hearing” any feedback from a clinician or family member regarding a cognitive impairment or behavioral limitation (Prigatano and Klonoff, 1998). They are not “indifferent” or apathetic in their emotional reactions to such discussions. In fact, they often propose counterarguments which may appear semi-logical (not bizarre in nature) to explain whatever impairments are brought to their attention (Prigatano et al., 2020). They can behave in a manner that actively discourages the examiner from asking further questions about their clinical status. While not displaying severe denial, Patient #2 acknowledged anxiety regarding her memory difficulties; her ability to acknowledge her distress would suggest “mild” denial. Thus the third screening item attempts to assess whether this feature is absent (see Figure 1).

Using the three-item scale listed in Figure 1, we suggest that if the total score is four or higher, denial may be an important contributing factor to the patient’s underreporting of their memory difficulties. This cutoff is suggested so that at least two of the three items are endorsed.

## Discussion

Screening for denial when studying anosognosia in individuals with MCI and early AD is important from a methodological perspective. By separating these phenomena, the strength of the biomarkers of anosognosia may be more clearly elucidated. For example the association of anosognosia with the DMN may show stronger correlations when patients showing denial are excluded from the research sample. Early neuroimaging research suggests that disturbances in metabolic activity in the medial prefrontal cortex (MPFC) and the posterior cingulate cortex (PCC) were related to impaired self-awareness in MCI (Johnson and Ries, 2010). Leech and Sharp (2014) reviewed the important role of the PCC in conscious awareness. Recent research has further suggested that a decline in the PCC network may be especially predictive of Alzheimer’s disease progression (Lee et al., 2020). By removing patients who show primarily denial of their memory impairments, the association of anosognosia with these structures may be more clearly understood.

Empirical efforts to describe the underlying psychological components of denial have revealed important findings. Ferrario et al. (2017) identified three basic dimensions. They are “denial of negative emotions,” “resistance to change,” and “conscious avoidance.” The “conscious avoidance” and “denial of negative emotions” are potentially captured by proposed screening Items #1 and #3. The patient can get angry with the clinician and/or significant other when attempts are made to directly talk about their impairment or limitations. They often try to avoid directly addressing the questions and pose counterarguments for why they do not “have a problem.” Note, Patient #2 explicitly stated that she was trying to avoid experiencing anxiety over her increasingly common memory failures.

The “resistance to change” component of denial is often encountered in psychotherapy or rehabilitation. Patients with classic anosognosia for hemiplegia do not resist any treatment efforts to help them improve (Prigatano et al., 2011). In contrast, those with moderate to severe traumatic brain injury (TBI) who demonstrate features of denial often resist the need to change their attitudes or behaviors (Prigatano and Klonoff, 1998).

Screening Item #2 attempts to rule out a common feature of anosognosia. In anosognosia, the person often appears perplexed by peoples’ emotional reactions to them since they do not perceive that what they have said or done may have upset someone. The presence of this skill, therefore, suggests the absence of anosognosia.

The diagnosis of MCI is a descriptive diagnosis (Peterson, 2004). Not all patients who have this diagnosis will advance to dementia. This is an important notion in patient care. If the patient has true anosognosia, then the progressive development of impaired self-awareness may, in fact, be a marker of a progressive dementing process (Gagliardi and Vannini, 2022; Prigatano and Russell, 2023).

## Limitations of this perspective

A prevailing problem in the study of impaired awareness of any cognitive and or behavioral ability following different brain disorders is the failure to separate denial from anosognosia. As noted above, this continues to be an important methodological issue in research, since non-neurological factors can contribute to an apparent lack of awareness. This paper does not solve that problem but attempts to emphasize its importance.

Second, we suggest a brief screening method that may be helpful in identifying patients who appear to be denying their deficits. We acknowledge that our scale has not been empirically validated but is proposed as a starting point when investigators wish to consider how this variable may influence research findings. If a patient shows probable denial on the proposed screening items, a more extensive assessment of denial may be useful when studying anosognosia in neurodegenerative disorders (e.g., the Illness Denial Questionnaire, Ferrario et al., 2017).

Finally, we acknowledge that our paper is a case study report and therefore has inherent limitations for generalizability and quantitative analysis.

## Data availability statement

The original contributions presented in the study are included in the article/supplementary materials, further inquiries can be directed to the corresponding author.

## Ethics statement

Written informed consent was obtained from the individual(s) for the publication of any potentially identifiable images or data included in this article.

## Author contributions

GP: Conceptualization, Methodology, Writing – original draft, Writing – review & editing. SR: Conceptualization, Data curation, Formal analysis, Writing – original draft. TM: Conceptualization, Writing – original draft, Writing – review & editing.
